# Coxsackievirus A11 is an immunostimulatory oncolytic virus that induces complete tumor regression in a human non-small cell lung cancer

**DOI:** 10.1038/s41598-023-33126-x

**Published:** 2023-04-12

**Authors:** Akira Sakamoto, Hiroyuki Inoue, Shohei Miyamoto, Shun Ito, Yasushi Soda, Kenzaburo Tani

**Affiliations:** 1 Laboratory of ALA Advanced Medical Research, Institute for Quantitative Biosciences, The University of Tokyo, Tokyo, Japan; 2 Department of Respiratory Medicine, Faculty of Medicine, Fukuoka University, Fukuoka, Japan; 3 Division of Oncology, Research Center for Medical Sciences, The Jikei University School of Medicine, Tokyo, Japan

**Keywords:** Non-small-cell lung cancer, Cancer therapy, Virology

## Abstract

Non-small cell lung cancer (NSCLC) is the leading cause of cancer-related mortality worldwide. Innovative treatment is required to improve overall survival rates for advanced NSCLC. Oncolytic virotherapy using enteroviruses has emerged as a promising anticancer strategy. To identify a novel, potent virotherapy with an improved safety profile, we assessed the oncolytic activity of 28 enteroviral strains and focused on coxsackievirus A11 (CVA11). CVA11 infection caused extensive oncolytic activity in all three of the examined human NSCLC cell lines, with high intercellular adhesion molecule-1 (ICAM-1) expression associated with greater CVA11-induced cytotoxicity. In vitro inhibition analysis using a pan-caspase inhibitor and western blot detection of cleaved poly (ADP-ribose) polymerase (PARP) indicated that apoptosis partly contributed to CVA11-driven cytotoxicity. CVA11 infection-induced immunogenic cell death in vitro was strongly suggested by substantial calreticulin expression and release of high mobility group box-1 protein (HMGB1). Moreover, in vivo treatment of human NSCLC xenografts with intratumoral CVA11 injection caused complete tumor regression in all treated mice, without significant weight loss. Our findings indicate that novel oncolytic virotherapy utilizing CVA11 may be less toxic and more effective than current treatments for human NSCLC, thus warranting further investigation in clinical trial settings, especially in combination with immunotherapy.

## Introduction

Lung cancer has a poor prognosis and is the leading cause of cancer-related mortality worldwide^1^. There are two main subtypes of lung cancer, namely small cell lung carcinoma and non-small cell lung cancer (NSCLC), which together make up 15% and 85% of all lung cancer cases, respectively. NSCLC is further classified as squamous cell carcinoma, adenocarcinoma, and large cell carcinoma, with 5-year relative survival rates of 14.3%, 13.3%, and 9.6%, respectively, in 2020^2^. The development of more effective anticancer treatments is required to improve overall survival in patients with advanced NSCLC.

In recent decades, oncolytic virotherapy using RNA viruses that specifically target tumor cells has emerged as a promising new strategy for various types of human cancers^3,4^. Since most RNA viruses replicate in the host cytosol without a DNA phase, RNA viruses are considered to be relatively safe and lack the genotoxicity caused by viral genome integration into the host DNA^5^. In particular, enteroviruses, which are members of the family Picornaviridae, are a diverse group of small RNA viruses that have been reported as novel oncolytic virotherapy candidates with immunostimulatory properties^6,7^. In this regard, they have several notable advantages. First, these viruses immediately induce robust cytolytic changes during cell-to-cell infection without harboring oncogenes that may lead to tumorigenesis. Second, reverse genetics systems can easily manipulate them to rescue positive-strand RNA viruses from complementary DNA. Importantly, most non-polio enteroviruses are very prevalent and are mainly associated with asymptomatic infection or mild disease^8^.

Previously, we performed a large-scale, two-step screening of 28 enteroviral strains and successfully determined that coxsackievirus B3 exhibited remarkable oncolytic cytotoxicity against nine human NSCLC cell lines, both in vitro and in vivo^9^. In addition, coxsackievirus A21 was reported to be a potent oncolytic enterovirus against tumor cells in multiple human cancers, such as breast cancer, bladder cancer, multiple myeloma, and malignant melanoma^10,11^. However, CVA21-infected mice developed lethal myositis with paralysis, which raised safety concerns^12^.

To establish a novel virotherapeutic agent with an improved safety profile, we focused on CVA11, which has low pathogenicity and broad oncolytic activity against solid cancers, as demonstrated by our screening of 28 enteroviruses (data not shown). We investigated the oncolytic activity of CVA11 towards human NSCLC cell lines both in vitro and in vivo. We also attempted to elucidate the viral cytocidal mechanism by investigating the mode of cell death and the antitumor immunogenic potential of NSCLC cells.

## Materials and methods

### Mice

Four-week-old female BALB/c nude mice were purchased from The Jackson Laboratory Japan (Yokohama, Japan). All animal experiments were approved by the Institutional Animal Care and Use Committee of the University of Tokyo, and carried out under the University of Tokyo guidelines for animal experiments, the Japanese government’s Law 105 Notification 6, regarding human cell culture, and the ARRIVE guidelines.

### Cell lines

NSCLC cell lines H1299 and H1975 were purchased from the American Type Culture Collection (ATCC) (Manassas, VA, USA). NSCLC cell line A549 was purchased from the RIKEN BioResource Research Center (Tsukuba, Japan). These cell lines were expanded for at least one passage before they were frozen at − 80 °C until use. Thawed cells were resuspended in Roswell Park Memorial Institute (RPMI) 1640 medium supplemented with 10% fetal bovine serum before they were plated on 150-mm-diameter dishes. Cells were incubated at 37 °C in a humidified atmosphere containing 5% CO_2_.

### Enterovirus production

CVA11 (the prototype Belgium-1 strain) was obtained from H. Shimizu (National Institute of Infectious Disease, Tokyo, Japan) and was propagated in H1299 cells. The 50% tissue culture infectious dose (TCID_50_) per milliliter on HeLa cell monolayers was determined as previously described^9^.

### Crystal violet staining

Cell viability was measured by crystal violet staining as previously described^13^. Adherent 1 × 10^5^ cells/well of H1299, 3 × 10^4^ cells/well of A549, and 1 × 10^5^ cells/well of H1975 cells on 24 well plates were infected with CVA11 at a multiplicity of infection (MOI) of 0.001, 0.01, 0.1, and 1 for 1 h, and the virus suspension was replaced with 500 μl of fresh medium. After 72 h, the cells were fixed with 0.5% glutaraldehyde in phosphate-buffered saline (PBS) for 15 min. Then, cells were washed with PBS and stained with 0.1% crystal violet solubilized in 2% ethanol-distilled water for 10 min. The leaving staining solution was removed and washed with PBS, and the plates were dried for 30 min.

### Neutralization assay

1 × 10^4^ cells/well of H1299 were sowed to 96 well plates and exposed to CD54 monoclonal antibody (Biolegend, San Diego, CA, USA) for 1 h. Subsequently, those cells were infected with CVA11 for 1 h at MOI of 0.001 or 0.01, replaced with fresh medium, and cultured for 48 h. Cell viability was confirmed by the CellTiter-Glo Luminescent Cell Viability Assay (Promega, Madison, WI, USA) following the manufacturer's instructions. Analysis was performed with the EnSpire Multimode Plate Reader (PerkinElmer, Waltham, MA, USA).

### CellTiter-Glo assay for cell viability

5–20 × 10^3^ cells/100 μl/ well of H1299 and 2 × 10^4^ cells/100 μl/well of H1975 cells were sowed to 96 well plates and incubated for 6 h. After that, those cells were infected with CVA11 for 1 h and replaced with fresh medium. The in vitro cell viability of CVA11-infected cells was confirmed with the CellTiter-Glo Luminescent Cell Viability Assay following the manufacturer's instructions. Analysis was performed with the EnSpire Multimode Plate Reader.

### Apoptosis inhibition assay

For our in vitro inhibition assay, 1 × 10^4^ cells/100 μl/well of H1299 cells sowed to 96 well plates were pretreated with the pan-caspase inhibitor z-VAD-fmk (R&D Systems, Minneapolis, MN, USA) (10 and 25 μM) for 1 h. After z-VAD-fmk was removed, H1299 cells were infected at MOI = 1 for 1 h. The virus suspension was removed, and 100 μl of fresh medium containing z-VAD-fmk (10 and 25 μM) was added to each well. After 24 h, cell viability was determined by CellTiter-Glo Assay following the manufacturer's instructions. Analysis was performed with the EnSpire Multimode Plate Reader.

### Flow cytometry analysis

NSCLC cell lines were incubated with a phycoerythrin-conjugated anti-human decay-accelerating factor (DAF) antibody (BD Biosciences, Franklin Lakes, NJ, USA), or an allophycocyanin (APC)-conjugated anti-human intercellular adhesion molecule-1 (ICAM-1) antibody (Biolegend). For surface calreticulin (CRT) analysis, both of the H1299 and H1975 cells infected with CVA11 (MOI = 1) for 12 h were subjected to flow cytometry analysis after cells were washed with FACS buffer (PBS with 3% fetal bovine serum) and fixed with 1% paraformaldehyde^14^. Data were collected using a FACS-Calibur (BD Biosciences) and analyzed using FlowJo software Ver 10.7.2 (Tree Star, Ashland, OR, USA).

### Annexin V staining

Following CVA11 infection against H1299 and H1975 cells for 12 h, apoptotic cells were resuspended in binding buffer (Hank's balanced salt solution with 2.5 mM Ca^2+^) and stained with APC-conjugated Annexin V (Biolegend) and 7-AAD (BD Biosciences). Data were collected using a FACSCalibur (BD Biosciences) after cells were washed with binding buffer and fixed with 1% paraformaldehyde and analyzed using FlowJo software Ver 10.7.2.

### Determination of HMGB1 level

The supernatant of H1299 cells infected with CVA11 (MOI = 1) for 6, 12, and 24 h were collected for detection of HMGB1 using the HMGB1 ELISA Kit II (Shino-Test Corporation, Tokyo, Japan), which was used according to the manufacturer's instructions. Briefly, diluted standards and samples were applied to microplate wells coated with anti-HMGB1 and incubated for 24 h at 37 °C. After washing five times with wash solution, the detection antibody was added to each well and incubated for 2 h at room temperature. Subsequently, wells were rewashed five times with wash solution, and substrate solution was added to each well with incubating for 30 min at room temperature. Finally, stop solution was added, and the absorbance of each well was measured to detect the HMGB1 concentration. Analysis was performed with the EnSpire Multimode Plate Reader.

### Western blotting

Protein samples were prepared by lysing CVA11 infected H1299 cells at MOIs of 0.001 and 1, and H1975 cells at MOIs of 0.1 and 1 with Cell Lysis Buffer (Cell Signaling Technology, Danvers, MA, USA) followed by SDS-PAGE was performed. Samples were loaded next to the molecular weight marker protein of Precision Plus Protein Dual Color Standards (Bio-Rad Laboratories, Hercules, CA, USA). After electrophoresis and transfer of proteins, they were targeted using primary antibodies against mixed lineage kinase domain-like protein (MLKL) (Cell Signaling Technology), phosphorylated MLKL (p-MLKL) (Cell Signaling Technology), poly (ADP-ribose) polymerase (PARP; Cell Signaling Technology), cleaved PARP (Cell Signaling Technology), and β-actin (Cell Signaling Technology). Subsequently, after the membrane was incubated with an HRP-linked Anti-rabbit secondary antibody (Cell Signaling Technology), SuperSignal West Dura Extended Duration Substrate (Thermo Fisher Scientific, Waltham, MA, USA) was used to detect protein bands. Analysis was performed using Images of western blotting of protein samples and molecular weight marker were respectively detected by chemiluminescently and visually using FUSION FX (Vilber, Paris, France).

### In vivo therapeutic studies

5 × 10^6^ cells of H1299 cells were injected subcutaneously into the left flanks of nude mice (day − 3), where they subsequently formed tumors. Three days after injection, the tumors were inoculated with CVA11 (1 × 10^6^ TCID_50_) once on day 0 or with the same dose of CVA11 on days 2, 4, 6, and 8. The tumor volume was calculated as (length × width × width)/2 and expressed as mean ± SEM. Animals were euthanized when the tumor volume exceeded 500 mm^3^.

### Statistical analyses

All statistical analyses were conducted using GraphPad Prism 7.0d software (GraphPad Software, San Diego, CA, USA). Data were analyzed by the two-tailed unpaired Student’s t-test, one-way ANOVA followed by Tukey’s multiple comparison test, or the nonparametric Mann–Whitney U-test. A *p*-value < 0.05 was considered statistically significant.

## Results

### CVA11 specifically killed human NSCLC cell lines

**Figure 1:**
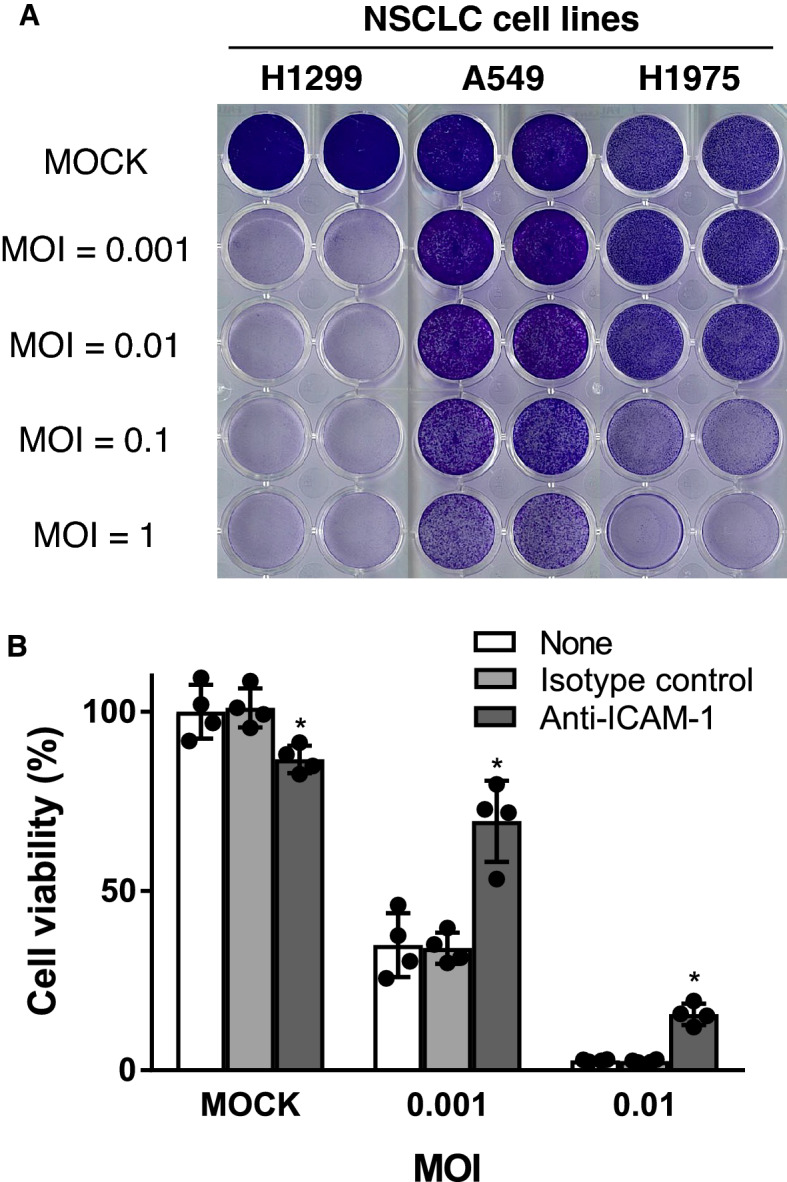
CVA11 preferentially killed human NSCLC cell lines. (**A**) The viability of three human NSCLC cell lines (H1299, A549, and H1975) due to CVA11-mediated cytotoxicity was confirmed by crystal violet staining after CVA11 infection at MOIs of 0.001, 0.01, 0.1, and 1 for 72 h. Live cells were stained violet. (**B**) The viability of H1299 cells due to CVA11-mediated cytotoxicity was confirmed by the Cell Titer-Glo Luminescent Cell Viability Assay after being treated with ICAM-1 neutralizing antibody and infected with CVA11 MOI of 0.001 and 0.01 for 48 h. The one-way ANOVA followed by Tukey’s multiple comparison test was performed (n = 4). (*) indicates *p* < 0.05 versus isotype control.

We first determined whether CVA11 infection induced oncolytic destruction of various human NSCLC cell lines. After NSCLC cell lines were infected with CVA11 at MOIs of 0.001, 0.01, 0.1, and 1, cell viability on day 3 post-infection was determined by crystal violet staining. Our results indicated that CVA11 infection led to extensive dose-dependent oncolytic activity in all three NSCLC cell lines (Fig. 1A). Indeed, both of H1299 and H1975 cells showed the highest cytotoxicity induced by CVA11 infection at MOIs of 0.001 and 0.1, respectively, while A549 cells were killed by CVA11 at MOI of 1.

### Human ICAM-1 was considered to be the cell surface receptor mediating CVA11 infection

Coxsackieviruses have recently been subdivided into distinct clusters within the Enterovirus genus based on genetic resemblance. Cluster C comprises coxsackie A virus serotypes 1, 11, 13, 15, and 17–22, 24^15^. Coxsackievirus A13 (CVA13), CVA18, and CVA21 have been shown to utilize ICAM-1 as their cellular receptor^16^, while other coxsackieviruses, such as coxsackievirus B3, have been shown to use DAF and coxsackievirus-adenovirus receptor protein (CAR)^17^. We therefore investigated the correlation between ICAM-1, DAF expression levels, and CVA11-mediated cytotoxicity in NSCLC cell lines.

Our results revealed that NSCLC cell lines with a high ICAM-1 level (H1299 and H1975) were susceptible to CVA11-mediated cytotoxicity, whereas DAF expression did not correlate with cytotoxicity (Table 1 and Fig. 1A). Furthermore, we used the CellTiter-Glo Assay to assess the effect of co-treatment with an ICAM-1 neutralizing antibody on the cell cytotoxicity induced by CVA11 infection in H1299 cells, which expressed a high ICAM-1 level on their surface. We found that addition of ICAM-1 neutralizing antibody significantly attenuated the CVA11 infection-triggered cytotoxicity compared to those with isotype antibody at MOI of 0.001 or 0.01 (Fig. 1B).

**Table 1:** Cell surface expressions of ICAM-1 and DAF in NSCLC cells.

Cell lines examined	Name	ICAM-1 (%)	DAF (%)
NSCLC	H1299	99.3	99.3
	A549	36.8	99.9
	H1975	99.9	99.8

These results suggested that CVA11 used ICAM-1 as one of the primary receptors for cell entry, leading to effective oncolysis.

### CVA11 cytotoxicity was time and concentration-dependent

**Figure 2:**
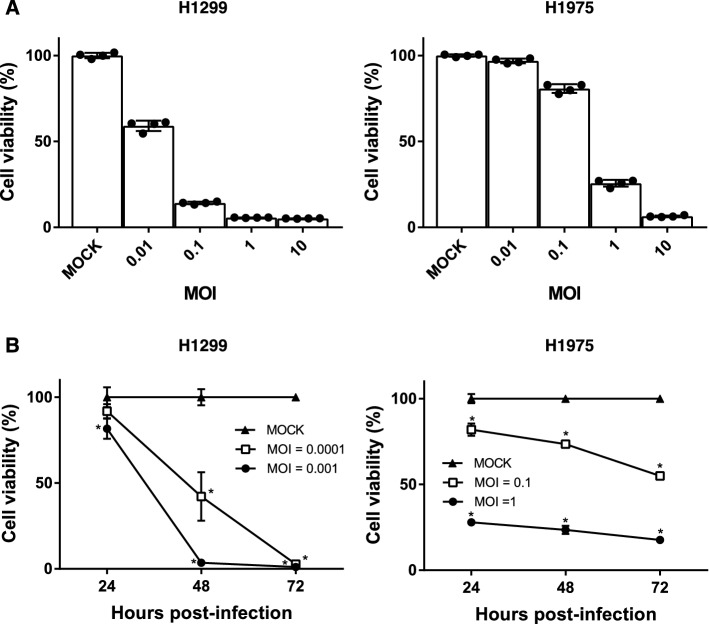
Cytotoxicity of CVA11 against NSCLC cell lines was MOI and time-dependent. (**A**,**B**) The cell viability of CVA11-infected H1299 and H1975 cells was observed using the Cell Titer-Glo Luminescent Cell Viability Assay at the indicated MOIs and times. (**A**) The MOI-dependent cytotoxicity of CVA11 against H1299 and H1975 cells was confirmed by setting MOI = 0.01, 0.1, 1, and 10 for 24 h. (**B**) The time-dependent cytotoxicity of CVA11 against H1299 and H1975 cells was confirmed by setting time = 24, 48, and 72 h with MOI = 0.0001 and 0.001 for H1299 cells and MOI = 0.1 and 1 for H1975 cells. The one-way ANOVA followed by Tukey’s multiple comparison test was performed (n = 4). (*) indicates *p* < 0.01 versus mock cells.

We subsequently analyzed cell viability using the CellTiter-Glo Assay to determine if cytotoxicity due to CVA11 infection was dependent on MOI, duration of infection, or both. First, we infected H1299 and H1975 cells with CVA11 at MOIs = 0.01, 0.1, 1, and 10, and found that cytotoxicity at 24 h increased with higher MOIs (Fig. 2A). Second, we infected CVA11 to H1299 cells at MOIs of 0.0001 and 0.001, and H1975 cells at MOIs of 0.1 and 1 every 24 h for up to 72 h, and showed that cytotoxicity increased with time (Fig. 2B).

### Cellular apoptosis machinery partly contributed to CVA11-mediated cytotoxicity

**Figure 3:**
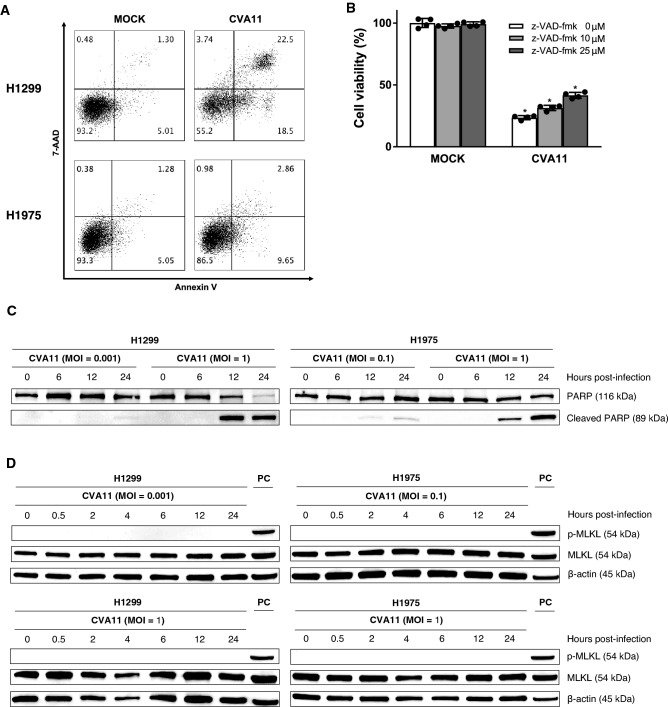
Correlation between caspase-dependent apoptosis and CVA11-mediated cytotoxicity in human NSCLC cells. (**A**) The early apoptotic population of H1299 and H1975 cells infected with CVA11 (MOI = 1) at 12 h post-infection was represented as percentages of annexin V-APC^+^/7-AAD^−^ cells analyzed by flow cytometry. (**B**) H1299 cells pretreated with dimethyl sulfoxide or z-VAD-fmk (10 and 25 μM) and incubated with mock virus or CVA11 (MOI = 0.1) were subjected to the CellTiter-Glo Luminescent Cell Viability Assay at 24 h post-infection. The one-way ANOVA followed by Tukey’s multiple comparison test was performed (n = 4). (*) indicates *p* < 0.01 versus mock cells. (**C**) Western blotting analysis of apoptosis-related proteins in CVA11-infected H1299 (MOI = 0.001 and 1) and H1975 (MOI = 0.1 and 1) cells. PARP and cleaved PARP bands are shown at 116 kDa and 89 kDa, respectively. (**D**) Western blot analysis of apoptosis-related proteins in CVA11-infected H1299 (MOI = 0.001 and 1) and H1975 (MOI = 0.1 and 1) cells. p-MLKL and MLKL bands are both shown at 54 kDa. HT29 cells treated with tumor necrosis factor-α (10 ng/mL), LCL-161 (100 nM), and z-VAD-fmk (20 μM) were used as a positive control (PC) for p-MLKL. Western blotting images were cropped, and full-length blots are included in Supplementary Fig. 1.

Evasion of apoptosis is fundamental to tumor initiation, progression, and maintenance. Despite the fact that many tumor cells have defective apoptosis-related decision-making machinery, these cells usually retain an intact cell-execution system^18^. Therefore, anticancer drugs that provide an effective apoptotic signal may successfully induce apoptosis in tumor cells. Hence, we examined whether CVA11 infection at MOI = 1 could induce apoptosis in H1299 and H1975 cells. Flow cytometry analysis showed that CVA11-infected H1299 and H1975 cells exhibited a high proportion of early apoptotic cells (annexin V^+^/7-AAD^−^) (Fig. 3A).

In H1299 cells, we further assessed the impact of pretreatment with z-VAD-fmk, a pan-caspase inhibitor that prevents apoptosis, on CVA11-driven cytotoxicity using the CellTiter-Glo Assay. CVA11-mediated cytotoxicity was significantly reduced by z-VAD-fmk in a dose-dependent manner (Fig. 3B).

In addition, we used western blot analysis to confirm the presence of cleaved PARP, a sensitive marker for apoptosis. CVA11 infected H1299 cells at MOIs of 0.001 and 1, and H1975 cells at MOIs of 0.1 and 1 incubated for 6, 12, and 24 h, and 89 kDa bands indicating cleaved PARP were detected (Fig. 3C). The findings demonstrated that the cleaved PARP protein was expressed in a time- and MOI-dependent fashion. Thus the apoptosis machinery contributed to CVA11-driven oncolysis and subsequent CVA11 replication in NSCLC cells.

Whereas, previous studies demonstrated that viral infection induced necroptosis also^19,20^. The implementation of necroptosis is dependent on the phosphorylation of MLKL^21^. So using western blotting, we checked a necroptosis protein, p-MLKL, with CVA11 infected H1299 cells at MOIs of 0.001 and 1, and H1975 cells at MOIs of 0.1 and 1. But as shown in Fig. 3D, we did not detect p-MLKL (54 kDa), while the result confirmed MLKL (54 kDa).

### CVA11 infection induced immunogenic cell death in NSCLC cell lines

Oncolytic viruses exert a persistent treatment effect not only through direct oncolysis, but also by inducing secondary immune responses against tumors^22^. The significant replication of these viruses within tumors results in immunological damage-associated molecular pattern (DAMP) signaling that increases immunogenicity within the tumor microenvironment^23^. Since previous studies demonstrated that several oncolytic viruses could induce adaptive antitumor immunity by generating tumor-specific cytotoxic T-lymphocyte responses^24^, we measured the expression of DAMPs such as CRT and HMGB1 to determine whether CVA11 infection could also cause immunogenic cell death.

**Figure 4:**
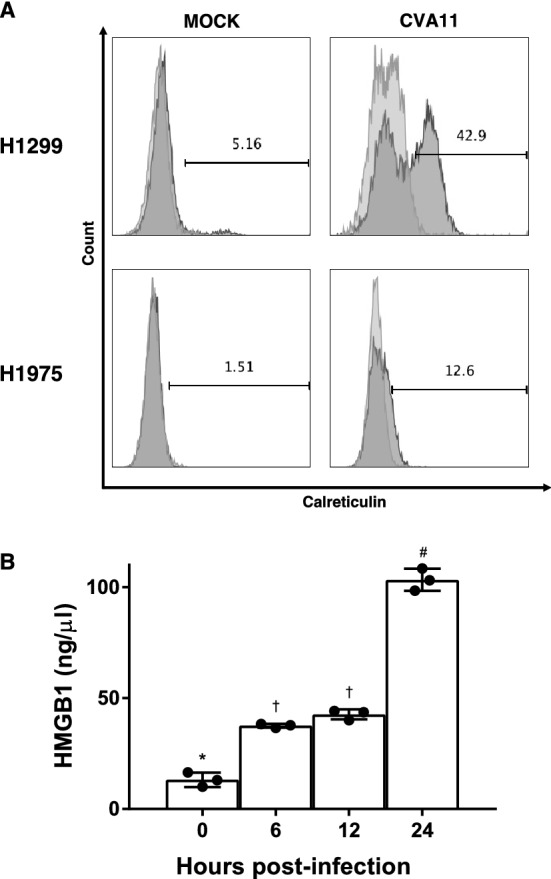
CVA11-infected NSCLC cells showed DAMP release indicative of immunogenicity. (**A**) CRT expression levels on the surface of H1299 and H1975 cells were determined by flow cytometric analysis 12 h after CVA11 infection (MOI = 1). (**B**) The released HMBG1 from H1299 cells infected with CVA11 (MOI = 1) and incubated for 6, 12, and 24 h was detected by ELISA. The one-way ANOVA followed by Tukey’s multiple comparison test was performed (n = 3). There are significant differences (*p* < 0.05) between the values indicated by each of the three symbols: *, †, and #.

Our flow cytometry analyses revealed that CVA11 infection at MOI = 1 induced abundant surface exposure of CRT on H1299 and H1975 cells (Fig. 4A), whereas mock treatment did not cause the same response. Furthermore, H1299 cells infected with CVA11 resulted in the time-dependent release of HMGB1, another determinant of immunogenicity (Fig. 4B).

### Serial intratumoral administration of CVA11 resulted in complete tumor regression in xenograft models of human NSCLC cells

**Figure 5:**
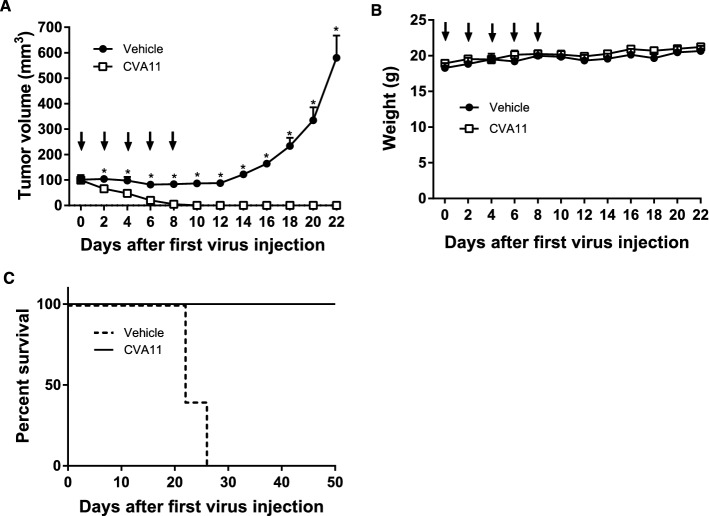
In vivo therapeutic effects of intratumoral CVA11 administration on subcutaneous human NSCLC xenografts. Schedule of CVA11 treatment: H1299 cells were injected subcutaneously into the left flanks of nude mice (day − 3). Three days after injection, H1299 xenograft-bearing mice were assigned to one of two groups (n = 5/group): untreated or treated with five serial intratumoral CVA11 injections on days 0, 2, 4, 6, and 8 (black arrows). (**A**) Tumor volumes are expressed as means ± SEM. The two-tailed unpaired Student’s t-test was performed. *, *p* < 0.05. (**B**) Body weights were measured on the indicated days. Data are shown as the mean ± SEM. The nonparametric Mann–Whitney U-test was performed. (**C)** Kaplan-Meier survival curves. The solid and dashed lines represent CVA11 and Vehicle, respectively.

We determined the in vivo oncolytic effect and tolerability of five consecutive intratumoral CVA11 injections in athymic nude mice bearing pre-established subcutaneous H1299 NSCLC xenografts. While these mice manifested complete tumor regression, untreated mice did not (Fig. 5A). Notably, none of the mice that received CVA11 exhibited significant body weight loss (Fig. 5B), and the survival rate until day 50 showed a trend of improvement in mice treated with CVA11 compared with untreated mice (Fig. 5C), illustrating the tolerability of CVA11 therapy.

## Discussion

Lung cancer is the leading cause of cancer deaths worldwide. In 2020, the number of lung cancer-related deaths was estimated at just under 1.8 million, which exceeded the combined number of deaths from breast, colon, and prostate cancers^1^. Novel anticancer therapies are necessary to improve the clinical outcomes of patients with this devastating disease. In this study, in vitro cytotoxicity assays identified CVA11 as a promising oncolytic virus against NSCLC (Figs. 1A, 2). Notably, our results that H1975 cells harboring L858R/T790M double epidermal growth factor receptor (EGFR)-tyrosine kinase mutations making them resistant to both first- and second-generation EGFR-tyrosine kinase inhibitors were susceptible to CVA11 infection leading to their cytolytic destruction (Fig. 1A) suggested its potential antitumor activity against refractory NSCLC patients with driver genetic abnormalities. Meanwhile, our findings demonstrated that CVA11 could induce marked oncolysis even at a low MOI = 0.001 in H1299, and that CVA11-induced cytotoxicity correlated positively with ICAM-1 expression levels in NSCLC cells (Table 1, Fig. 1B). ICAM-1 therefore might be a primary receptor for CVA11 entry into cancer cells. Previous reviews implicated ICAM-1 in the progression, prognosis, and metastasis of solid tumors, including lung cancer^25,26^. Our findings thereby form the basis for using CVA11 to treat patients with advanced NSCLC in particular.

We also found that CVA11 induced robust caspase-mediated apoptosis and substantially contributed to cytolysis and viral replication in human NSCLC cells (Fig. 3). Since cancer cells in general are notorious for their resistance to chemotherapy^27^, the ability of CVA11 to provoke apoptosis is important for treating patients with refractory NSCLC.

Furthermore, we sought to confirm whether necroptosis was a cause of cell death triggered by CVA11 infection. Previous studies demonstrated that viral infection induced necroptosis. We hypothesized that CVA11 infection would induce not only apoptosis but also necroptosis in NSCLC cell lines, and we performed western blot analysis to measure inducible levels of p-MLKL, which is related to necroptosis. Contrary to our expectations, CVA11 infection did not induce necroptosis. Li, Z. et al. reported that coxsackievirus A16, a similar virus with CVA11, activated caspase 8^28^, which was previously reported as the inhibitor of necroptosis by Fritsch, M. et al^29^. It could be speculated that the cytotoxicity induced by CVA11 infection might mainly related to apoptosis machinery during oncolysis.

The potent and persistent antitumor effects of oncolytic viruses may result from antitumor immunity induced by virus replication-mediated destruction of cancer cells, which leads to antigen spreading^30^. Mechanistically, secondary antitumor immunity likely depends on the quantity of inflammatory DAMPs within shrinking tumors^9^. Therefore, it is important to determine whether oncolytic viruses can cause immunogenic cell death. This study confirmed that CVA11 infection induced immunogenic changes such as CRT exposure and HMGB1 release in NSCLC cells (Fig. 4).

In addition to the direct oncolytic activities of CVA11, its immunostimulatory properties during the innate immunity phase may subsequently promote the effective generation of adaptive T cell immunity through robust stimulation of antigen-presenting cells such as dendritic cells and macrophages, which engulf newly released tumor-associated antigens and neoantigens. In fact, Xiang et al. reported in mouse model that the combination of the oncolytic adenovirus with the anti-PD-1 antibody in lung cancer mice model achieved synergistic antitumor efficacy^31^. This finding suggested the possibility that the combination of CVA11 with immune-checkpoint inhibitors might improve the antitumor efficacy of immunotherapy by altering the tumor microenvironment of non-immunogenic (“cold”) tumor types.

An important outcome of this study is that serial intratumoral CVA11 administration completely eliminated subcutaneous H1299 xenografts with favorable tolerability. This remarkable antitumor activity may be due to the abundant expression of ICAM-1 in H1299 cells. ICAM-1 may therefore be a predictive biomarker for the efficacy of CVA11 treatment. In terms of toxicity, no mice in any experiment died or manifested body weight loss, while other group B coxsackieviruses, including coxsackievirus B5, have been reported to induce spastic paralysis, myocarditis, and viral replication in the mouse central nervous system^32^. ICAM-1 is constitutively expressed at low levels in various cell types, including fibroblasts, endothelial cells, and leukocytes, pathologically expressed in NSCLC tumors at higher levels that correlate positively with those of soluble lCAM-1 in serum, and associated with metastatic disease^33^. CVA11 treatment may therefore be a safer therapeutic modality for patients with advanced NSCLC than other enterovirus strains, although further preclinical studies would be required to investigate safety profiles in more detail.

In conclusion, we found that CVA11 is a promising oncolytic virus for the treatment of patients with advanced NSCLC, and that it acts by inducing apoptosis and has immunostimulatory properties. We believe that these characteristics are promising for the future preclinical and clinical development of CVA11 therapies, especially in combination with cancer immunotherapy.

## Supplementary Information

Supplementary Information.

## Data Availability

The datasets used and analyzed during the current study are available from the corresponding author upon reasonable request.
