# Supplementary material for: Coxsackievirus A11 is an immunostimulatory oncolytic virus that induces complete tumor regression in a human non-small cell lung cancer
Source: Sci Rep. 2023 Apr 12;13:5924. doi: 10.1038/s41598-023-33126-x (PMC10097657; doi:10.1038/s41598-023-33126-x)
Supplement: Supplementary file 1 — Supplementary Information. [file 41598_2023_33126_MOESM1_ESM.pdf]

Coxsackievirus A11 is an Immunostimulatory Oncolytic Virus that Induces Complete Tumor Regression in a Human Non-Small Cell Lung Cancer

Akira Sakamoto, Hiroyuki Inoue, Shohei Miyamoto, Shun Ito, Yasushi Soda and Kenzaburo Tani.

a

PARP (MW = 116 kDa)

H1299

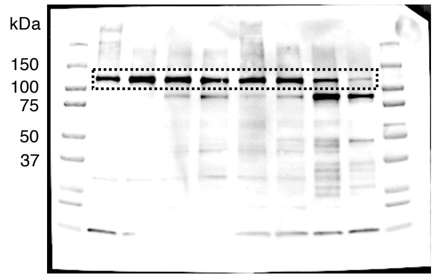

H1975

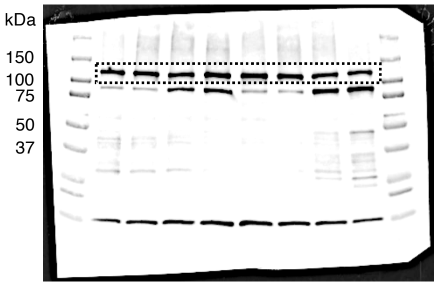

Cleaved PARP (MW = 89 kDa)

H1299

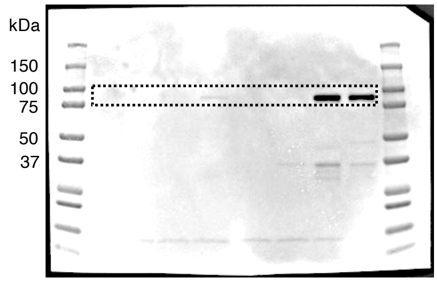

H1975

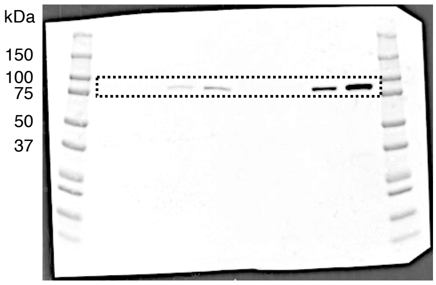

b

H1299

p-MLKL (MW = 54 kDa)

MOI = 0.001

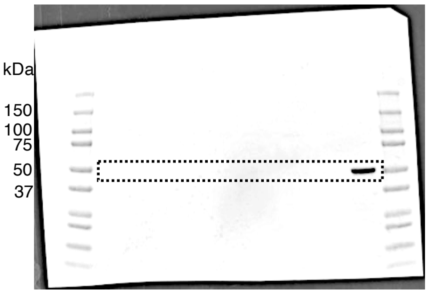

MOI = 1

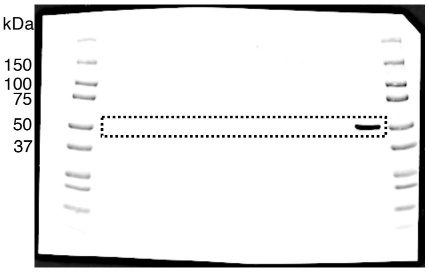

MLKL (MW = 54 kDa)

MOI = 0.001

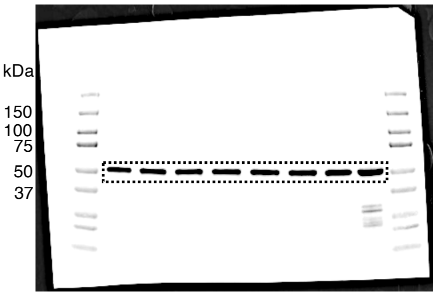

MOI = 1

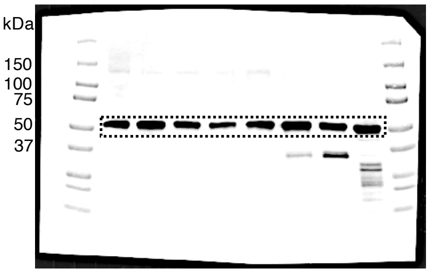

β-actin (MW = 45 kDa)

MOI = 0.001

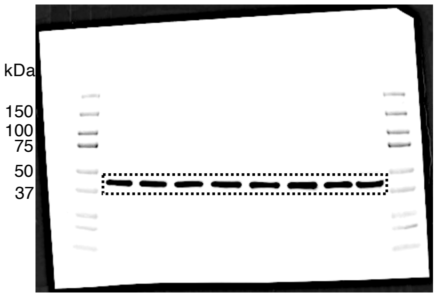

MOI = 1

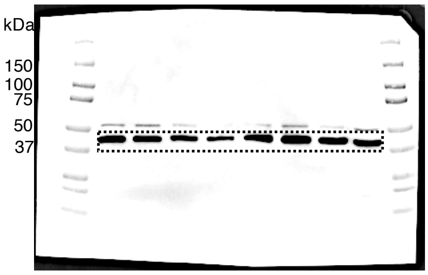

c

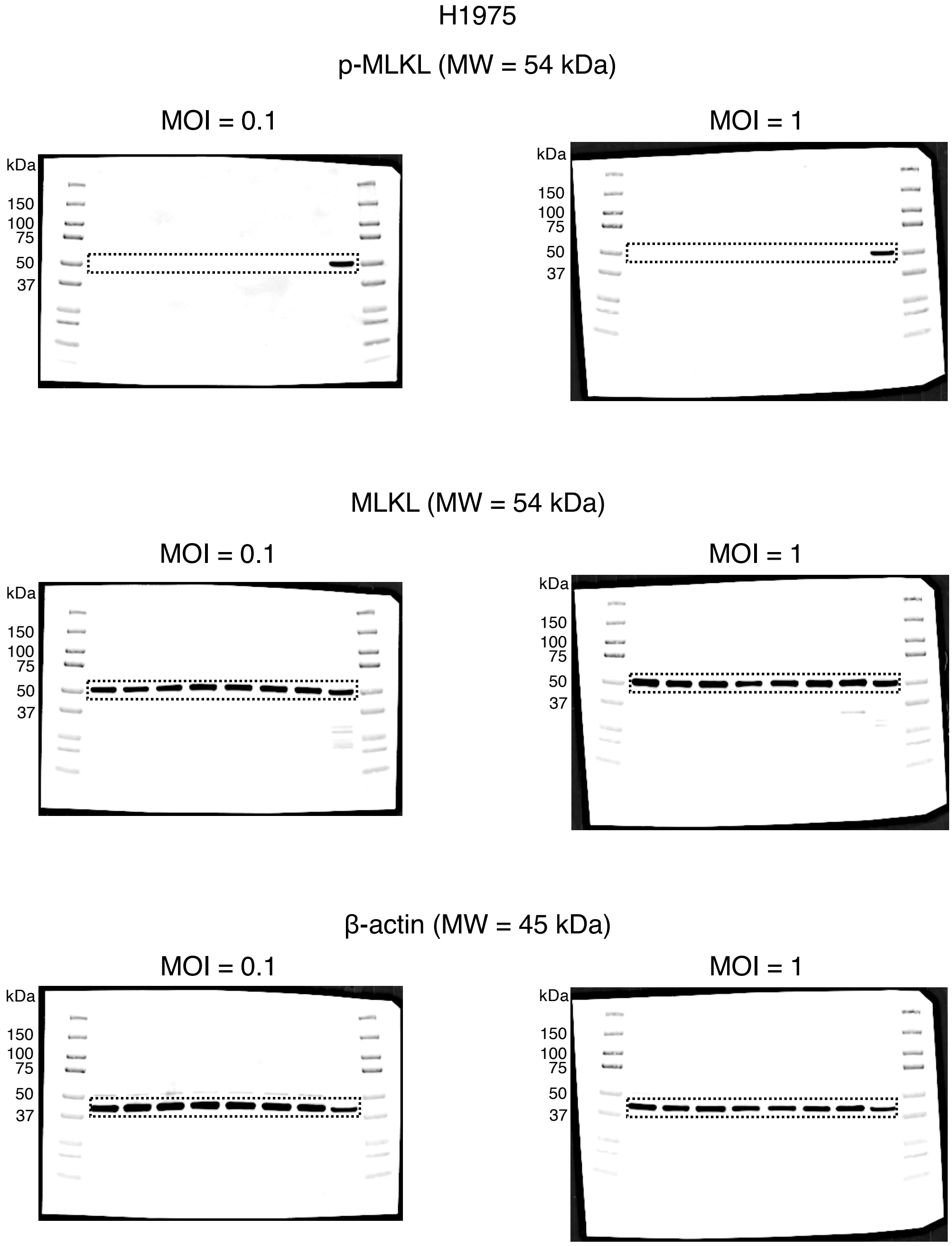

Supplementary Figure 1. Western blotting full-length images of a, b, and c are related to Fig. 3C (H1299 and H1975), Fig. 3D (H1299), and Fig. 3D (H1975), respectively. Relevant field is boxed in dashed line. Both ends of each membrane are the molecular weight marker protein of Precision Plus Protein Dual Color Standards, which ranged from 10 kDa to 250 kDa.
